# The therapeutic response in Gorham’s syndrome to the beta-blocking agent propranolol is correlated to VEGF-A, but not to VEGF-C or FLT1 expression

**DOI:** 10.1186/s13104-015-1259-9

**Published:** 2015-08-04

**Authors:** Jessica Baud, Abderrahim Lomri, Denis Graber, Andreas Bikfalvi

**Affiliations:** INSERM U1029, Allée Geoffroy St. Hilaire, 33 615 Pessac, France; Université Bordeaux, Allée Geoffroy St. Hilaire, 33 615 Pessac, France; Service de Pédiatrie, Hôpital Saint Louis, Rue du Dr Schweitzer, 17019 La Rochelle Cedex, France

**Keywords:** Gorham’s syndrome, VEGF, Angiogenesis, Lymphangiogenesis

## Abstract

**Background:**

Gorham’s syndrome is a rare illness of unknown etiology. It is characterized by a local proliferation of blood or lymphatic vessels that in bones leads to progressive resorption and destruction. The cause of the disease is not elucidated, and therapeutic options remain limited.

**Case presentation:**

We report herein the case of a young female Caucasian patient aged 18 years with diffuse Gorham syndrome. In tissue specimens angiogenesis and massive lymphangiogenesis as well as the expression of vascular endothelial growth factor-A (VEGF-A) and neuropilins was observed. Lymphangiogenesis is a prominent feature of the disease and a number of lymphatic markers were found to be expressed, however only VEGF-A, but not vascular endothelial growth factor-C (VEGF-C) was found to be elevated in the circulation. Circulating levels of soluble VEGF receptor-1 were also not elevated. Furthermore, the patient responded favorably and the disease was stabilized following treatment with the beta-blocking agent Propranolol alone which acts on VEGF-A alone, but not on soluble VEGF receptor-1 levels.

**Conclusion:**

This suggests that the disease is dependent on VEGF-A, but on neither VEGF-C, the major driver of lymphangiogenesis, nor FLT1. Furthermore, Propranolol acts on VEGF-A but not FLT1 expression.

**Electronic supplementary material:**

The online version of this article (doi:10.1186/s13104-015-1259-9) contains supplementary material, which is available to authorized users.

## Background

Gorham’s syndrome is a rare illness of unknown etiology. It is characterized by a local proliferation of blood or lymphatic vessels that leads to progressive resorption and destruction in bones [1]. The lesions consist of numerous thin walled vascular or lymphatic channels in association with massive osteolysis. The lesions may extend into adjacent bones, viscera or soft tissue, and may be accompanied by local fibrosis. The cause of the disease is not elucidated. The therapeutic options to treat this condition are still limited.

The use of Propranolol has been reported previously in infantile hemangioma [2], pediatric lymphatic malformation [3] and in Gorham’s syndrome [4, 5]. Vascular endothelial growth factor-A (VEGF-A) was previously shown to be increased in Gorham’s syndrome, and Propranolol to be able to decrease VEGF-A levels [5, 6]. However, the full spectrum of VEGF family members and, in particular, vascular endothelial growth factor-C (VEGF-C) and vascular endothelial growth factor receptor-1/fms-like tyrosine kinase-1 (VEGFR1 or FLT-1) has not been investigated and no definite explanation for the therapeutic effect was given.

## Case presentation

We report herein the case of young Caucasian female patient of 18 years of age with diffuse Gorham’s syndrome. Surprisingly, since lymphangiogenesis is a prominent feature in the disease, VEGF-A but not VEGF-C was found to be elevated in the patient’s circulation. Circulating levels of soluble FLT-1 were also not elevated. Furthermore, the patient responded favorably to the β-blocking agent Propranolol which acts on VEGF-A, but not on FLT-1 levels.

**Figure 1:**
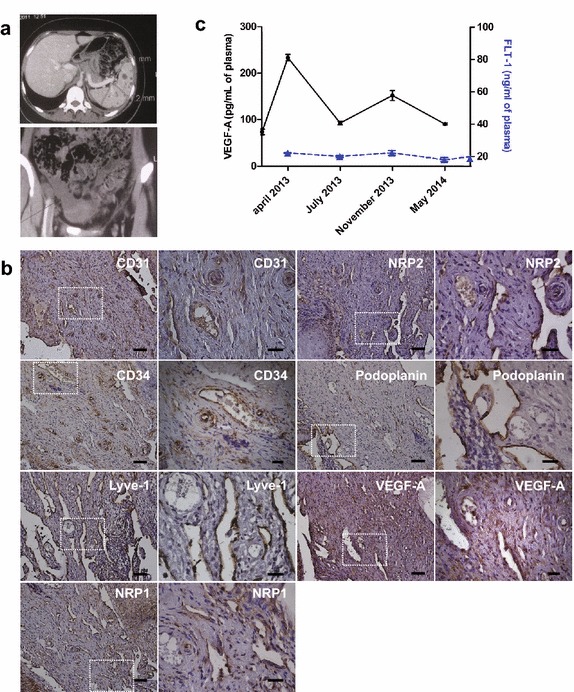
**a** Radiological images of spleen (*upper image*) and pelvic region (*lower image*) at diagnosis. Multiple hypodense nodules are present in the spleen and fluid accumulation in the pelvic region is seen. **b** Immunohistochemistry of cervix tissue for blood and lymphatic vessel markers. Blood vessels were revealed by cluster of differentiation 31 and 34 (CD31, CD34), neuropilin-1 (NRP1, *arterial marker*), neuropilin-2 (NRP2, *venous marker*) immunostaining. Lymphatics were detected by Lymphatic vessel endothelial hyaluronan receptor-1 (LYVE-1) and Podoplanin immunostaining. Blood and lymphatic vessels are detected both by CD31 and NRP2 immunostaining. Furthermore, VEGF-A was significantly expressed in the tissue. Areas in the *dotted squares* are shown at higher magnification in the right hand images for each marker. *Scale bars*: *Left panels* 50 µm, *right panels* 20 µm). **c** Detection of vascular endothelial growth factor-A (VEGF-A) and soluble fms-like tyrosine kinase (FLT-1) in plasma samples by ELISA. VEGF-A (*black dots*) and FLT-1 (*blue triangle*). We noticed a decrease in VEGF-A levels between July 2013 and May 2014 (after Propranolol treatment) compared to April 2013 (before treatment). No significant change was observed for FLT-1. Vascular endothelial growth factor-C (VEGF-C) was below detection limit in all samples. Control levels of healthy donors were 75.78 pg/ml and 20.58 ng/ml for VEGF-A and FLT-1 respectively (pooled sample, n = 6).

Clinical symptoms were already present at the age of 9. At diagnosis at the age of 15, the patient exhibited pronounced diffuse lesions in bone and soft tissue (Figure 1a). Furthermore, this was accompanied by severe gynecological symptoms with loss of chylous/lymphatic liquid. Biopsies of cervical lesions revealed pronounced angiogenesis and lymphangiogenesis (Figure 1b). VEGF-A was strongly expressed at the site of the lesion along with neuropilin 1 (NRP1) and 2 (NRP2) (Figure 1b). Plasma VEGF-A levels were found to be increased by threefold in comparison to a pool (n = 6) of young healthy female donors (control 75.13, versus 233.42 pg/ml for the patient). VEGF-A levels before treatment were higher than those reported in breast cancer patients (136.22 ± 9.95 pg/ml) [7]. The CARE Checklist (2013) of information is included in Additional file 1.

Because of the strong lymphangiogenic component found in the biopsy specimen, we hypothesised that VEGF-C levels would be highly up-regulated. However, in contrast to VEGF-A, we found that circulating VEGF-C levels were not increased, and were below the limit of. Furthermore, circulating FLT-1 (average: 20.58 ng/ml) was not increased (Figure 1c).

The fact that pronounced lymphangiogenesis is associated with elevated VEGF-A levels is in agreement with the observations of Halin et al. [8]. They have shown that inflammation-induced lymphangiogenesis is potently blocked by systemic administration of a vascular endothelial growth factor (VEGF)-A neutralizing antibody and is, thus, dependent on VEGF-A.

In contrast to our results, Ozeki et al. [3] have reported that Plasma VEGF-A, C, and D levels are significantly higher in patients with lymphatic malformation in comparison to control samples. Lymphatic malformation and Gorham’s syndrome are not the same clinical entities which may explain the differences observed.

Treatment with Propranolol LP (2 mg/kg/day) was initiated. The patient responded well to this treatment with a disappearance of gynecological symptoms and stabilization of bone lesions. VEGF-A levels dropped after 3 months of treatment to nearly control levels. At 7 months of treatment, some increase in VEGF-A levels was observed which subsequently decreased again after 12 months of treatment. The rebound effect at 7 months may be due to transient treatment interruption by the patient since the heart rate increased during that period.

Morimoto et al. [5] have demonstrated that a combination treatment including surgery, interferon and Propranolol is able to improve the clinical evolution of Gorham’s syndrome. Furthermore, they showed with a single measurement (time point) that the combination of surgery, interferon and Propranolol™ is able to decrease VEGF-A levels. In opposition to these results, we demonstrate that Propranolol alone is able to improve clinical outcome and decreases VEGF-A levels measured at several time points. Soluble FLT1 could also explain the therapeutic effect because soluble FLT1 is an endogenous inhibitor of VEGF-A. However, soluble FLT1 is not up-regulated after Propranolol treatment. Nir et al. [4] described an effect of Propranolol on clinical outcome, but the study did not include measurement of VEGF family members or other angiogenic factors.

## Conclusions

Our results suggest that, despite pronounced lymphangiogenesis, Gorham’s syndrome seems to be dependent on VEGF-A and not on VEGF-C or FLT-1. Furthermore, this case demonstrates that Propranolol can be safely administered and has striking therapeutic efficacy as a single agent in Gorham’s syndrome by decreasing circulating VEGF-A but not by modifying VEGF-C or FLT-1 levels.

## Additional file

Additional file 1: CARE Checklist (2013) of information to include when writing a case report.

## Abbreviations

CD31: cluster of differentiation 31
CD34: cluster of differentiation 34
LYVE-1: lymphatic vessel endothelial hyaluronan receptor-1
VEGF: vascular endothelial growth factor
VEGFR: vascular endothelial growth factor
FLT1: fms-like tyrosine kinase-1
NRP: neuropilin
